# Changes in evidence for studies assessing interventions for COVID-19 reported in preprints: meta-research study

**DOI:** 10.1186/s12916-020-01880-8

**Published:** 2020-12-17

**Authors:** Theodora Oikonomidi, Isabelle Boutron, Olivier Pierre, Guillaume Cabanac, Philippe Ravaud, the COVID-19 NMA Consortium

**Affiliations:** 1 Université de Paris, UMR 1153 CRESS Inserm, 75004 Paris, France; 2 Clinical Epidemiology Unit, Hôtel-Dieu Hospital, Assistance Publique-Hôpitaux de Paris, (AP-HP), 75004 Paris, France; 3 Cochrane France, Hôtel-Dieu Hospital, 75004 Paris, France; 4 University of Toulouse, IRIT UMR 5505 CNRS, 118 route de Narbonne, 31062 Toulouse Cedex 9, France; 5 Department of Epidemiology, Mailman School of Public Health, Columbia University, New York, NY USA

**Keywords:** Preprint, Meta-research, COVID-19, Coronavirus

## Abstract

**Background:**

The increasing use of preprints to disseminate evidence on the effect of interventions for the coronavirus disease 2019 (COVID-19) can lead to multiple evidence sources for a single study, which may differ in the reported evidence. We aim to describe the proportion of evidence on the effect of interventions for COVID-19 from preprints and journal articles and map changes in evidence between and within different sources reporting on the same study.

**Methods:**

Meta-research study. We screened the Cochrane living systematic review and network meta-analysis (COVID-NMA) database to identify all preprints and journal articles on all studies assessing interventions for COVID-19 published up to 15 August 2020. We compared all evidence sources (i.e., preprint and associated journal article) and the first and latest versions of preprints for each study to identify changes in two evidence components: study results (e.g., numeric change in hazard ratio, odds ratio, event rate, or change in *p* value > or < 0.05 in any outcome) and abstract conclusions (classified as positive, negative or neutral regarding the intervention effect, and as reporting uncertainty in the findings or not). Changes in study results were further classified as important changes if they (1) represented a change in any effect estimate by ≥ 10% and/or (2) led to a change in the *p* value crossing the threshold of 0.05.

**Results:**

We identified 556 studies. In total, 338 (61%) had been reported in a preprint: 66 (20%) of these had an associated journal article (median time to publication 76 days [interquartile range (IQR) 55–106]) and 91 (27%) had > 1 preprint version. A total of 139 studies (25% of the overall sample) were reported in multiple evidence sources or versions of the same source: for 63 (45%), there was a change in at least one evidence component between or within sources (42 [30%] had a change in study results, and in 29 [21%] the change was classified as important; 33 [24%] had a change in the abstract conclusion). For studies with both a preprint and an article, a median of 29% (IQR 14–50) of total citations were attributed to the preprint instead of the article.

**Conclusions:**

Results on the effect of interventions for COVID-19 are often reported in multiple evidence sources or source versions for a single study. Evidence is not stable between and within evidence sources. Real-time linkage of all sources per study could help to keep systematic reviews up-to-date.

## Background

The coronavirus disease 2019 (COVID-19) pandemic has created a need for rapid performance and dissemination of systematic reviews on the effectiveness of preventive, therapeutic, and post-acute care interventions to guide clinical practice, policy-making, and guideline development [1]. Researchers compiling systematic reviews are faced with the challenge of synthesizing large amounts of evidence disseminated rapidly, principally through two channels of communication: preprint servers and journals [2].

Systematic reviews traditionally rely on peer-reviewed journal articles, which represent the final form of a study report, and may only undergo changes through retraction or publication of a correction. However, in the context of COVID-19, many researchers have chosen to disseminate their findings by using preprints (i.e., non-peer-reviewed manuscripts, accessible on open archives). Preprints offer several benefits. First, they are published rapidly. For example, publication on medRxiv, the preprint server for the health sciences, takes only 5 days [3]. In comparison, publication in a peer-reviewed biomedical journal can take several months. Second, preprints can easily be updated as additional data are obtained and analyzed, by uploading a new version of the manuscript on the preprint platform. Ultimately, preprint servers help disseminate evidence at the rapid pace imposed by the COVID-19 pandemic.

However, the use of preprints can lead to multiple evidence sources (i.e., the preprint and the associated journal article) as well as multiple versions of the same evidence source (i.e., the first and updated preprint versions) being available for a single study. Important information, such as outcome effect sizes, could undergo changes between and within these sources as evidence for a single study evolves over time. Multiplicity of incongruent evidence sources can mislead systematic reviewers and other research end-users.

In this study, we aimed to (1) describe the proportion of evidence on the effect of interventions for COVID-19 from preprints and journal articles, (2) map changes in evidence for the same study between different sources (i.e., preprint and journal article) and between different versions of the same source (i.e., preprint versions), and (3) describe how results from each source are disseminated by using the citation count, Altmetric Attention Score, and PubPeer comments.

## Methods

This meta-research study is ancillary to the Cochrane living systematic review and network meta-analysis (NMA) on COVID-19 (hereafter COVID-NMA) (https://covid-nma.com/, PROSPERO CRD42020182600) [4]. COVID-NMA is a living systematic review, in which all available evidence related to COVID-19 is continuously collected, critically appraised, and synthesized using pairwise comparison and NMA methods. The protocol for the present study can be provided upon request by the corresponding author.

Our sample comprises all studies assessing preventive, therapeutic, or post-acute care interventions for COVID-19 that were published as preprints or journal articles up to 15 August 2020.

### Data sources

To identify eligible studies, we screened the COVID-NMA database, which provides an exhaustive archive of COVID-19 studies assessing the effect of interventions. As part of the COVID-NMA project, medRxiv, and PubMed are searched daily, independently by two reviewers, to identify eligible studies. Secondary sources, such as the Living OVerview of Evidence (L·OVE) database, are searched as a quality control. The search strategy is in Additional file 1: Methods S1.

### Eligibility criteria and study selection

We included all preprints and journal articles indexed in the COVID-NMA database, from inception to 15 August 2020, with the following study designs: randomized controlled trials (RCTs), observational studies (i.e., case series, case-control, cohort, interrupted time series, studies modeling the effects of population-level interventions). We excluded case series with < 5 participants, systematic reviews, prognosis and diagnosis studies, and modeling studies using simulation methods. We included reports on preventive, therapeutic, and post-acute care interventions, for healthy individuals, patients with COVID-19, or recovered individuals, respectively. Because the scope of the COVID-NMA is already very broad, studies assessing Traditional Chinese Medicine and other types of Traditional, Complementary and Alternative Medicine (TM/CAM) interventions were excluded [4]. Studies not performed with humans were also excluded.

Two reviewers (T.O. and O.P.) independently screened all records. Disagreements were resolved by consensus or by consultation with a senior reviewer (I.B.).

### Linking all evidence sources for each study

We sought to link preprints and journal articles reporting results from the same study. For this purpose, we used a four-step approach:

1. We performed a systematic search of preprints and published articles as part of the COVID-NMA (as described above). We used the same eligibility criteria for preprints and articles. One author (T.O.) downloaded these data in Excel format and used three ways to identify potential preprint-article pairs: (a) by using the Excel search function to identify articles for which the name of the article author matched that of the first author of an included preprint, and comparing the titles of potential matches to verify that they reported the same study; (b) by extracting the intervention type assessed in each preprint and article and comparing the titles of preprints and articles that assessed the same intervention to identify pairs that reported the same study; and (c) by comparing the trial registration number for preprints and articles reporting RCTs.
2. We performed additional searches of the Dimensions academic search engine (https://app.dimensions.ai). We used the name of the first author of each preprint to identify articles by the same author in any authorship position. We did this by downloading the Dimensions dataset in Excel format, filtering entries that were not journal articles, and using the search function to search for the name of the preprint’s first author in the column containing all article author names. When a potential preprint–article pair was identified in this manner, we compared the titles and the names of the remaining authors to decide whether the two sources reported findings from the same study.
3. One author (G.C.) designed an algorithm that relies on preprint metadata obtained via the Crossref API (https://github.com/CrossRef/rest-api-doc) (e.g., author ORCIDs, author names, sequence of coauthors listed in the bylines, title words, preprint/publication timelines) to identify journal articles reporting on the same study as the included preprints. All links identified by the algorithm were then reviewed by one author (T.O.) to remove false-positives. Furthermore, the accuracy of the algorithm was validated by comparing its results for 740 preprint–journal article pairs, against all links established by the medRxiv platform as of 14 July 2020. The algorithm correctly linked 99.73% of the 740 pairs, with only 2 false-negatives. Detailed results on the accuracy of the algorithm will be reported in a validation study [Cabanac et al., unpublished data].
4. Finally, we contacted the corresponding author of each preprint that was classified as unpublished in the above two steps via email (see Additional file 1: Methods S2 for a template) to ask whether the preprint had been accepted for journal publication. For preprints that had not been accepted, we asked whether they had been submitted to a journal, or if the authors intended to submit them in the future. We sent a reminder email to authors who did not respond to our first email. We received 123 responses from the 272 authors contacted (45% response rate). No new preprint–journal article links were identified in this step.

In this report, we include preprint–journal article links identified up to 2 September 2020.

### Data extraction

We extracted the following general characteristics of studies: study design, intervention, country of affiliation for the corresponding author, and data sharing (i.e., whether the authors were willing to share the dataset used in the study or not, from the data sharing statement).

#### Characteristics of evidence sources

We classified each source as a preprint or journal article. For journal articles, we manually extracted whether the journal had issued corrections or a retraction notice by searching the journal website and Retraction Watch (https://retractionwatch.com/retracted-coronavirus-covid-19-papers/). We used an algorithm to automatically extract the number of versions on the preprint server for each preprint and the date of online publication for each preprint and journal article.

#### Changes between and within evidence sources

We identified all studies in our sample for which there was more than one evidence source (i.e., a preprint and a journal article) or more than one version of the same evidence source (i.e., > 1 preprint version).

First, we sought to compare important evidence components *between* the following evidence sources:

- First preprint version versus journal article
- Latest preprint version versus journal article (if > 1 preprint versions were available)

Second, we sought to compare evidence components *within* evidence sources (the first preprint version versus the latest preprint version, if > 1 versions were available).

To perform these comparisons, we downloaded all abovementioned sources for each study in portable document format (PDF). We entered each pair of files in PDF Converter (Enterprise 8). This software allows users to compare documents by automatically detecting and highlighting changes in text (words added or deleted between versions). We reviewed the highlighted text to identify changes in the following evidence components, which may affect systematic reviewers’ appraisal of the effect of the intervention assessed in the study:

1. Change in any study result. We searched for numeric changes in at least one of the following effect-size metrics: hazard ratio, odds ratio, relative risk, event rate, correlation or regression coefficient (for country-level studies), or in the statistical significance (i.e., *p* value changed from > to < 0.05, or vice versa), for any outcome. To characterize the magnitude of change in results, we considered the change to be important if (1) it represented an increase or decrease by ≥ 10% of the initial value in any effect estimate and/or (2) it led to a change in the *p* value crossing the threshold of 0.05. For evidence source pairs that had a change in results, we additionally extracted whether the sample size had changed among or between evidence sources. The sample size was defined as the number of individuals enrolled in the study or the number of countries/regions analyzed (for population-level studies of policy interventions).
2. Change in the study conclusion reported in the abstract. First, we assessed the conclusion to determine two aspects:
  If the abstract conclusion was positive, neutral, or negative regarding the effect of the intervention (i.e., whether the authors focused on improvement in any efficacy outcome or reduction in harms; versus lack of impact on any outcome; versus focus on deterioration of efficacy or safety outcomes).
  If the abstract conclusion reported uncertainty in the findings (i.e., whether the authors emphasized the need for additional studies to confirm the findings and/or used mild phrasing such as “might be effective” versus strong phrasing such as “These results prove the efficacy of the intervention”).

We considered any change in conclusion among positive, neutral, or negative or change between reporting versus not reporting uncertainty to constitute change in the conclusion.

#### Dissemination of evidence sources

To describe the dissemination of the different evidence sources, we used an algorithm designed by one of the authors (G.C.) to automatically extract the following usage data for each preprint–journal article pair: citation count, Altmetric Attention Score (extracted from the Dimensions database) [5], and the number of PubPeer comments (extracted from the PubPeer Application Programming Interface [API]). These metrics were selected because they represent evidence dissemination by different end-user groups. Citations reflect use of an evidence source in academic communications. The Altmetric Attention Score tracks the occurrences of a source mentioned in the media and online bibliographic reference managers [6]. Finally, PubPeer data reflect attention by researchers in the form of crowdsourced peer review. Commenters can write comments or ask for clarifications about the study and the study authors can post a reply [7]. Usage data were last updated on 21 October 2020.

#### COVID-NMA subgroup

We sought to identify changes in evidence in a subgroup of studies currently being used for quantitative evidence synthesis in the COVID-NMA. These are RCTs and non-randomized studies that are interrupted time series or non-randomized studies using causal inference analysis or multivariable regression adjustment, with ≥ 150 incident users.

In addition to the study results and conclusion, we searched for changes in the following methodologic components that could affect the appraisal of risk of bias [8, 9]: blinding of participants, clinicians, or outcome assessors; amount and handling of missing data; randomization process and allocation concealment (in RCTs); inclusion of participants in the study or the analysis; and statistical method used to adjust for confounders and confounders adjusted for (in non-randomized studies).

One reviewer (T.O.) extracted data for all included studies by using a structured, piloted form (see Additional file 1: Methods S3). A second reviewer (O.P.) independently extracted data from all sources for 20% of the studies for all variables except for assessment of the conclusion, which was duplicated in full. Disagreements were resolved by consensus or by consulting a senior reviewer (I.B.). The agreement between reviewers was > 80% for all variables.

### Data analysis

We used descriptive statistics to describe study characteristics and changes in evidence. We summarized data usage (e.g., citation count) as median and interquartile range (IQR). Because our sample included evidence sources published over an 8-month period, we accounted for the difference in exposure time by dividing each usage metric by the number of days since the date of first online publication.

## Results

**Fig. 1:**
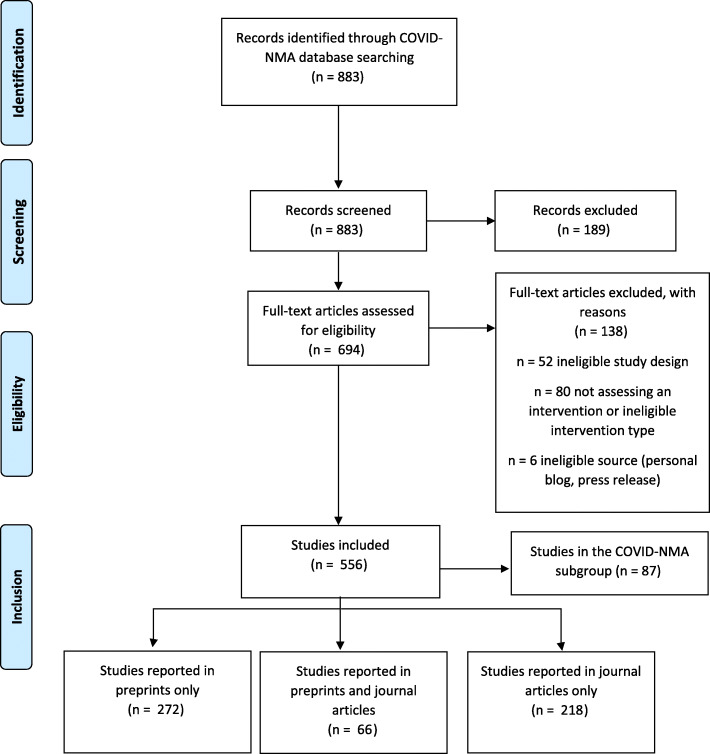
Flow chart

We identified 556 studies assessing interventions for COVID-19 (Fig. 1). The most commonly assessed interventions were social distancing/lockdown policy measures (*n* = 90, 17%) and antimalarial agents (*n* = 76, 14%) (Table 1). In half of the studies, the country of affiliation for the corresponding author was China (*n* = 144, 26%) or the USA (*n* = 135, 24%).

**Table 1:** Characteristics of 556 studies assessing interventions for COVID-19 prevention, treatment or rehabilitation

Characteristics	***n*** = 556
**Intervention (%)**^**a**^	
Social distancing, lockdown, or travel restrictions	90 (17)
Antimalarials	76 (14)
Monoclonal antibodies	52 (10)
Corticosteroids	37 (7)
Other^b^	288 (53)
**Country of affiliation for the corresponding author (%)**	
China	144 (26)
USA	135 (24)
Italy	39 (7)
France	32 (6)
UK	26 (5)
Other^c^	180 (32)
**Data sharing (%)**	
Data will be shared upon request to the authors	186 (33)
Publicly available dataset	108 (19)
No data sharing	62 (11)
Unclear or unreported	200 (36)
**Has a peer-reviewed evidence source (%)**	**284 (51)**
**Evidence source (%)**	
Preprint	272 (49)
Journal article	218 (39)
Preprint plus journal article	66 (12)
**Status of studies initially published as preprints**	***n*** **= 338**
Published in a journal (%)	66 (20)
Accepted for publication (%)	2 (0)
Unpublished, submitted to a journal	107 (32)
Unpublished, will be submitted to a journal	9 (3)
Unpublished, will not be submitted to a journal	5 (1)
Unpublished, unknown status (no response from authors)	149 (44)

^a^May not add up to 100% because of rounding

^b^Other interventions include health care organization, respiratory support, combination antivirals, antiretrovirals, angiotensin-converting enzyme inhibitor/angiotensin II receptor blocker, convalescent plasma, personal protective equipment, non-specific antiviral, broad-spectrum antiviral, anticoagulant, gas inhalation, advanced therapy medicinal products, organ support, population testing, antibiotics, non-steroidal anti-inflammatory drugs, respiratory stimulants, antiparasitic, immunosuppressants, kinase inhibitors, radiation, rehabilitation calcium channel blockers, and vaccination

^c^Other countries include Abu Dhabi, Albania, Australia, Belgium, Brazil, Brunei, Canada, Chile, Cuba, Denmark, Germany, Greece, Hong-Kong, India, Iran, Ireland, Israel, Japan, Jordan, Malaysia, Mexico, New Zealand, Norway, Portugal, Qatar, Saudi Arabia, Singapore, Slovakia, Spain, South Africa, South Korea, Sri Lanka, Switzerland, Thailand, The Netherlands, Tunisia, Turkey, Ukraine, and Vietnam. All < 4% of total sample

### Characteristics of evidence sources in the overall sample

**Fig. 2:**
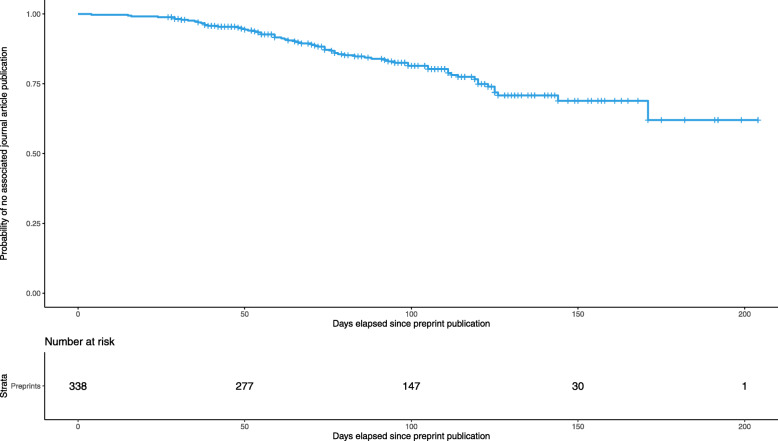
Survival plot depicting the probability of a preprint remaining unpublished as journal article for 338 studies initially published as preprints. This survival plot presents the probability that a study initially published as a preprint remains unpublished as a journal article. The *x*-axis represents the number of days elapsed since the preprint first appeared online. Of the 338 studies published as preprints from the onset of the COVID-19 pandemic to 15 August 2020, 66 had an associated journal article by 2 September 2020

Overall, 61% (*n* = 338) of all studies had been reported in a preprint (Table 1). The remaining 39% (*n* = 218) were directly published as journal articles. Among the 338 studies reported as preprints, 66 (20%) had then been published as journal articles. The median delay from publication of the first preprint version to publication of the associated journal article was 76 days (IQR 55–106) (Fig. 2).

We obtained additional information about the status of the 272 unpublished preprints by contacting the authors. We received responses for 123 preprints: 2 had been accepted for publication in a journal, 107 (32%) had been submitted for publication, and 9 (3%) will be submitted. A small proportion of authors (*n* = 5, 1%) did not intend to submit the preprint to a journal (Table 1).

Regarding the update of evidence over time, for 27% (*n* = 91) of preprints, > 1 version was uploaded to the preprint server (median number of versions per preprint: 1 [IQR 1–2, range 1 to 6]), and 2% of journal articles had been updated (*n* = 1 retracted, *n* = 4 had a published correction).

#### Changes between and within evidence sources in the overall sample

We found multiple sources of information for 139 (25%) studies: 66 had a preprint and a journal article and 91 had > 1 preprint version (18 studies had both a journal article and > 1 preprint version). For 45% (*n* = 63) of studies, we identified changes in at least one evidence component between or within evidence sources for the same study (Table 2). In total, 30% (*n* = 42) of studies had changes in study results and 24% (*n* = 33) had changes in the abstract conclusion. Regarding important changes in results (i.e., changes representing an increase or decrease by ≥ 10% of the initial effect estimate and/or leading to a change in *p* value at the 0.05 threshold), 21% (*n* = 29) of studies had important changes in study results (Additional file 1: Table S1). Among the total 54 changes in results observed between and within all evidence pairs, 18 (33%) evidence pairs also had a change in sample size.

**Table 2:** Change in evidence components, between and within evidence sources, in the overall sample and in a subgroup of randomized controlled trials and observational studies usable for quantitative evidence synthesis^a^

	Overall sample (***n*** = 139)^**b**^				COVID-NMA subgroup (***n*** = 25)^**a**^			
	Change between or within at least one evidence source pair (***n*** = 139)	First to latest preprint version (*n* = 91)	First preprint version to journal article (*n* = 66)	Latest preprint version to journal article (*n* = 18)	Change between or within at least one evidence source pair (***n*** = 25)	First to latest preprint version (*n* = 18)	First preprint version to journal article (*n* = 15)	Latest preprint version to journal article (*n* = 7)
**Change in at least 1 evidence component**	**63 (45)**	**35 (38)**	**36 (55)**	**8 (44)**	**15 (60)**	**8 (44)**	**12 (86)**	**3 (43)**
Change in study results	**42 (30)**	23 (25)	25 (38)	6 (33)	**10 (40)**	5 (28)	9 (64)	3 (43)
Change in risk of bias assessment	**–**	–	–	–	**5 (20)**	1 (6)	4 (27)	1 (14)
Change in abstract conclusion	**33 (24)**	18 (20)	19 (29)	3 (17)	**8 (32)**	5 (28)	5 (36)	1 (14)

^a^Includes studies used in quantitative evidence synthesis and GRADE development in the COVID-NMA project (randomized controlled trials; interrupted time-series, non-randomized studies using causal inference analysis or multivariable regression adjustment, including at least 150 incident users), with multiple evidence sources or evidence source versions. For more information see: https://covid-nma.com/emulated/

^b^Includes the studies in the COVID-NMA subgroup

**Fig. 3:**
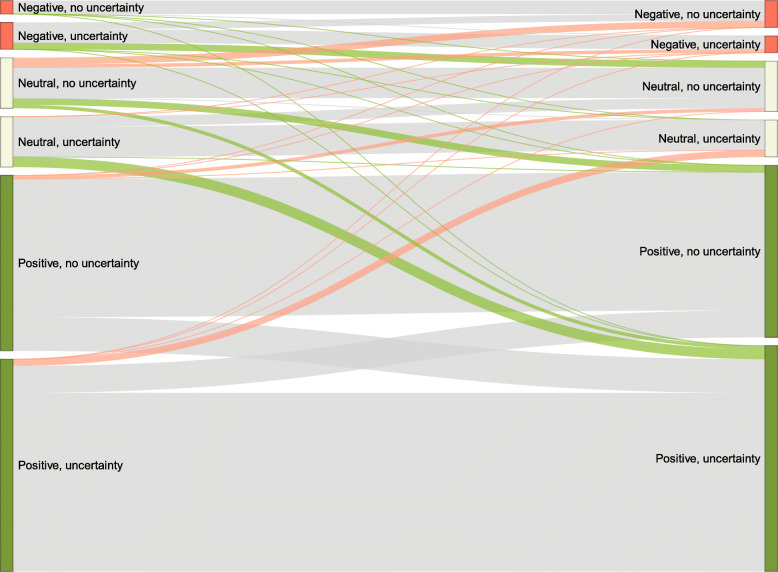
Direction of changes in the abstract conclusion of 139 studies assessing preventive, treatment, or post-acute care interventions for COVID-19, from the first preprint version to the associated journal article or latest preprint version of each study. This Sankey diagram presents the changes made to the abstract conclusion for the same study, between the first preprint version on the left, and the latest available source version on the right (journal article or latest preprint version for studies that have not been published in a journal). The colored flow lines represent the direction of the change: green lines represent change from negative to neutral or positive, or from neutral to positive, and red lines represent change from positive or neutral to negative, and from positive to neutral

Of all changes in conclusion (*n* = 40 changes; some studies had changes both between and within evidence sources), the most common change was from positive without reporting uncertainty, to positive with reporting of uncertainty (*n* = 12). In terms of changes among positive, neutral, and negative, the most common changes were from neutral to positive (*n* = 6) (Fig. 3).

### Characteristics of evidence sources in the COVID-NMA subgroup

There were 87 studies in the COVID-NMA subgroup, which consists of RCTs and observational studies included in the Cochrane living systematic review and is available online to support decision-makers. Overall, 64% (*n* = 56) had been reported in a preprint, 25% (*n* = 14) had then been published as journal articles, and 36% (*n* = 31) were directly published as journal articles. The median delay from the publication of the first preprint version to publication of the associated journal article was 74 days (IQR 36–97). For 30% of preprints, > 1 version was uploaded to the preprint server (median number of versions per preprint: 1 [IQR 1–2, range 1 to 4]). One journal article had been retracted and one had a published correction.

#### Changes between and within evidence sources in the COVID-NMA subgroup

In total, 25 studies of the COVID-NMA subgroup had multiple evidence sources or multiple versions of the same source (Table 2). We identified changes in any evidence component, between any evidence source, for 15 (60%) studies (Table 2). Changes were identified most frequently in study results (40%, *n* = 10, with any change in results, and 20%, *n* = 5, with an important change in results). Five studies (20%) had changes in methodologic components that may affect risk of bias assessment and 8 (32%) had changes in the abstract conclusion.

Finally, we identified the following changes that may affect risk of bias assessment: (1) addition of information about the propensity score model of non-randomized studies, such as additional confounders entered in the model, and specification of the propensity score being time-dependent (*n* = 2); (2) reporting of additional information about the conduct of the study that indicates prevalent user bias (*n* = 1); and (3) addition or removal of information about allocation concealment in RCTs (*n* = 2).

### Dissemination of evidence sources

Usage data for preprint–journal article pairs are presented in Additional file 1: Table S2 and Additional file 1: Figure S1. More preprints than articles received 0 citations (*n* = 10 of 65 [usage data could not be retrieved for 1 preprint], 15%, and *n* = 3 of 66, 5%, respectively). Of the total citations for each study, a median of 29% (IQR 14–50) was attributed to preprints.

A similar proportion of preprints and articles had an Altmetric Attention score of 0 (*n* = 4, 6% for both preprints and articles). Furthermore, we examined the breakdown of the Altmetric Score for the 66 preprint–journal article pairs. Specifically, we examined their score in the Policy documents domain, which reflects the citations a report has received in policy documents. Of the 66 preprint–journal article pairs, 9 preprints (14%) and 10 articles (15%) had a score > 0 in the Policy documents domain (median score for preprints 0, IQR 0–0; median score for articles 0, IQR 0–0).

Finally, 92% of preprints and 95% of journal articles had 0 PubPeer comments. For both the preprints and journal articles with PubPeer comments, the median number of comments was 0 (IQR 0–0).

## Discussion

This meta-research study showed that results for most studies assessing the effect of interventions for COVID-19 were initially disseminated in preprints, and the delay in publication of these results in journal articles takes 2 months. One-third of studies disseminated as preprints had at least one update since they first appeared online, with the addition of either more preprint versions or associated journal articles. Nearly half of these studies had changes in at least one important evidence component between or within evidence sources.

Preprints make a vital contribution to the body of evidence on the effect of interventions for COVID-19. The delay between preprint publication and journal article publication is 76 days (IQR 55–106); hence, for several months, preprints constitute the main source of evidence for a study. However, preprints constitute “living evidence” that is not stable. This flexibility allows researchers to keep up with evolving datasets (e.g., in studies exploring the association between policy interventions and mortality rates) and disseminate findings from interim analyses. Therefore, it is possible to observe changes in the reported evidence among sources for the same study, even though both sources were accurate at the time of redaction. For example, the first version of a preprint may accurately report the findings of an interim analysis of an RCT, before participant enrollment has been completed. The final version of the preprint, reporting the analysis of the complete dataset could have a different sample size and different results. The flexibility of preprints also allows authors to correct potential errors in the report following crowd-sourced peer review (e.g., in the form of PubPeer comments). One consequence of this flexibility is that evidence changes can lead to misleading results of systematic reviews. Systematic reviewers need to be aware of updates in the evidence reported in preprints in order to update the review accordingly. One such example is the ELACOI RCT [10]. The first preprint version of the ELACOI RCT reported findings for 44 participants, with 24% of those receiving lopinavir/ritonavir experiencing adverse events [10], but the final version of the preprint, published on medRxiv 3 weeks later, reported findings for 86 participants, with 35% of those receiving lopinavir/ritonavir experiencing adverse events. The authors did not specify that they were reporting preliminary results in the first preprint version.

To our knowledge, only one other study assessed changes in preprint–article pairs of studies of COVID-19 of any design [11]. The authors found changes that strengthened or softened the data and abstract conclusions in 27% of a randomly selected sample of COVID-19 preprint–article pairs. Our results are in agreement, although they are not directly comparable, because we focused on studies assessing interventions.

### Meaning of the study

Our findings point to a pressing need for systematic reviewers to monitor the literature for updates so as to use the latest evidence source for each study. This task is near impossible to perform manually (e.g., 6480 preprints were hosted on medRxiv on 1 September 2020) [12]. Despite efforts by preprint servers to alert users when a preprint is published in a journal, these may not be optimal during the pandemic. For example, of the 61 medRxiv preprint–journal article pairs we identified, medRxiv displayed the linkage for only 26 on 2 September 2020.

The large-scale use of preprints is a novel development for the biomedical sciences [13], and new mechanisms to manage this evidence dissemination track need to be invented. The algorithm designed for the present study offers such a mechanism by detecting the appearance of new evidence sources in real-time and summarizing this information in a web interface [14].

### Strengths and weaknesses of the study

This is the first study to assess changes between evidence sources for studies evaluating interventions for COVID-19. First, we used a large database to capture all relevant evidence sources published during the first semester of the pandemic. Second, we assessed changes in important evidence components that may affect the results of systematic reviews and thereby affect decision-making. Third, we elicited information about the publication status of preprints by contacting authors. Finally, we designed an algorithm that links preprints to their associated journal articles and flags the appearance of new preprint versions.

Our study has limitations. First, we extracted usage data soon after publication, and a different amount of time had elapsed from the publication of each study to retrieval of usage data. However, preprints tend to receive the majority of views near the time of upload on the server [11]. Additionally, we present usage data normalized by the number of days since publication for each study. Second, we retrieved usage data from the Dimensions database. Had we used a different source, we may have obtained different figures, owing to the different data sources used by each database [15].

## Conclusions

The results of studies assessing the effect of interventions for COVID-19 are often initially disseminated in preprints. The delay in publication of these results in journal articles was > 2 months, so preprints are a key evidence source for systematic reviews. Studies disseminated as preprints are often reported in multiple preprint versions and in associated journal article, and the evidence reported across these information sources is not stable over time. This situation could affect the results of systematic reviews and thus clinical and policy decision-making. Real-time linkage of all evidence sources for the same study is needed to avoid decision-making based on outdated evidence.

## Supplementary Information

**Additional file 1: Methods S1-S3**, **Tables S1-S2**, **Figure S1**. **Methods S1**. Search strategy used in the COVID-NMA database. **Methods S2** – Email template used to contact preprint authors. **Methods S3**. Data extraction form. **Table S1**. Sensitivity analysis of change in evidence components using important change in study results. **Table S2**. Usage data for preprint–journal article pairs. **Figure S1**. Distribution of usage data in preprint-journal article pairs.

## Data Availability

The datasets generated and/or analyzed during the current study will be made publicly available on https://zenodo.org after this report has been published.

## Abbreviations

COVID-19: Coronavirus disease 2019
RCT: Randomized controlled trial
NMA: Network meta-analysis
PDF: Portable document format
API: Application programming interface
IQR: Interquartile range
