# Supplementary material for: Changes in evidence for studies assessing interventions for COVID-19 reported in preprints: meta-research study
Source: BMC Med. 2020 Dec 17;18:402. doi: 10.1186/s12916-020-01880-8 (PMC7745199; doi:10.1186/s12916-020-01880-8)
Supplement: Supplementary file 1 — Additional file 1: Methods S1-S3, Tables S1-S2, Figure S1. Methods S1. Search strategy used in the COVID-NMA database. Methods S2 – Email template used to contact preprint authors. Methods S3. Data extraction form. Table S1. Sensitivity analysis of change in evidence components using important change in study results. Table S2. Usage data for preprint–journal article pairs. Figure S1. Distribution of usage data in preprint-journal article pairs. [file 12916_2020_1880_MOESM1_ESM.docx]

**Additional file 1**

**Methods S1: Search strategy used in the COVID-NMA database**

**Methods S2: Email template used to contact preprint authors**

**Methods S3: Data extraction form**

**Table S1: Sensitivity analysis of change in evidence components using important change in study results**

**Table S2: Usage data for preprint–journal article pairs.**

**Figure S1: Distribution of usage data in preprint-journal article pairs**

**Methods S1: Search strategy used in the COVID-NMA database**

The COVID-NMA uses a multi-step search strategy of primary and secondary data sources. The protocol describing this process has been reviewed and approved by Cochrane (i.e., Cochrane Emergency and Critical Care, proof in process).

1. PubMed (<https://pubmed.ncbi.nlm.nih.gov>)

The search strategy for PubMed was developed with Robin Featherstone, Information Specialist, at the Cochrane Editorial & Methods Department. The following search strategy is used to identify randomized trials:

| Search | Query |
| --- | --- |
| #9 | #8 Filters: **Publication date from 2020/01/01** |
| #8 | Search: **#4 AND #7** |
| #7 | Search: **#5 NOT #6** |
| #6 | Search: **animals[mh] NOT humans[mh]** |
| #5 | Search: **randomized controlled trial[pt] OR controlled clinical trial[pt] OR randomized[tiab] OR placebo[tiab] OR drug therapy[sh] OR randomly[tiab] OR trial[tiab] OR groups[tiab]** |
| #4 | Search: **#1 OR #2 OR #3** |
| #3 | Search: **severe acute respiratory syndrome coronavirus 2[Supplementary Concept]** |
| #2 | Search: **COVID-19[Supplementary Concept]** |
| #1 | Search: **"2019 nCoV"[tiab] OR 2019nCoV[tiab] OR "2019 novel coronavirus"[tiab] OR "COVID 19"[tiab] OR COVID19[tiab] OR "new coronavirus"[tiab] OR "novel coronavirus"[tiab] OR "SARS CoV-2"[tiab] OR (Wuhan[tiab] AND coronavirus[tiab])** |

The following search strategy is used to identify observational studies:

| **Search** | **Query** |
| --- | --- |
| #9 | #8 Filters: **Publication date from 2020/01/01** |
| #8 | Search: **#6 NOT #7** |
| #7 | Search: **animals[mh] NOT humans[mh]** |
| #6 | Search: **#4 NOT #5** |
| #5 | Search: **editorial[pt] OR comment[pt] OR letter[pt] OR newspaper article[pt]** |
| #4 | Search: **#1 OR #2 OR #3** |
| #3 | Search: **severe acute respiratory syndrome coronavirus 2[Supplementary Concept]** |
| #2 | Search: **COVID-19[Supplementary Concept]** |
| #1 | Search: **"2019 nCoV"[tiab] OR 2019nCoV[tiab] OR "2019 novel coronavirus"[tiab] OR "COVID 19"[tiab] OR COVID19[tiab] OR "new coronavirus"[tiab] OR "novel coronavirus"[tiab] OR "SARS CoV-2"[tiab] OR (Wuhan[tiab] AND coronavirus[tiab])** |

1. MedRχiv (<https://www.medrxiv.org>): A curated list of records on COVID-19 and SARS-CoV-2 is available at <https://connect.biorxiv.org/relate/content/181>. Note that this list also includes sources listed in bioRχiv, but only the sources published on MedRxiv are screened (i.e., titles in blue rather than red).
2. LitCOVID (<https://www.ncbi.nlm.nih.gov/research/coronavirus/>): All studies listed under “treatment” are screened.
3. Secondary sources:
   1. The L.OVE platform (<https://app.iloveevidence.com/loves/5e6fdb9669c00e4ac072701d>:

The L.OVE platform provides a collection of studies assessing interventions for COVID-19. These studies are identified by using the following comprehensive search strategy:

coronavir* OR coronovirus* OR betacoronavir* OR "beta-coronavirus" OR "beta-coronaviruses" OR "corona virus" OR "virus corona" OR "corono virus" OR "virus corono" OR hcov* OR "covid-19" OR covid19* OR "covid 19" OR "2019-ncov" OR cv19* OR "cv-19" OR "cv 19" OR "n-cov" OR ncov* OR (wuhan* and (virus OR viruses OR viral)) OR sars* OR sari OR (covid* and (virus OR viruses OR viral)) OR "severe acute respiratory syndrome" OR mers* OR "middle east respiratory syndrome" OR "middle-east respiratory syndrome" OR "covid-19-related" OR "2019-ncov-related" OR "cv-19-related" OR "n-cov-related"

The LOVE platform applies this search strategy to all major databases, including PubMed, EMBASE and LitCovid. The COVID-NMA team screens the COVID-19 collection of interventional studies provided by L.OVE in its entirety.

- 1. The Cochrane COVID-19 Study register (<https://covid-19.cochrane.org/>): The search strategies for the Cochrane register are available at <https://community.cochrane.org/about-covid-19-study-register>. All searches for the Cochrane register underwent peer-review.
  2. The Retraction Watch Database for retracted studies (<https://retractionwatch.com/retracted-coronavirus-covid-19-papers/>).

**Methods S2: Email template used to contact preprint authors**

Dear Dr [Last name],

We are a team of researchers led by Professors Isabelle Boutron and Philippe Ravaud from the Université de Paris and Cochrane France. We were very interested to read the report of your recent study, [Study title], published as a preprint. We salute you for the development and completion of this important research project undertaken in the most challenging of times.

We would like to **include your preprint in a meta-epidemiologic study**we are conducting, ancillary to our ongoing project [Covid-NMA](https://covid-nma.com/). Our aim is to describe the use of preprints for the dissemination of evidence during the Covid-19 pandemic. **We kindly request that you respond to the questions below** by replying to this e-mail.

1.Have you submitted this preprint article to a journal for publication? (Please respond Yes or No)

2.If **No**, do you intend to submit it in the future? (Please respond Yes or No)

3. If **Yes**, how many times have you submitted it for publication so far? ____
3.1. What is the date of the first time you submitted it for publication? ___/___/2020
3.2. If you have submitted it more than once, what is the date of the last time you submitted it for publication? ___/___/2020

Finally, we would be grateful if you could let us know **when this preprint article is accepted** in a journal, and when it is published.

Please do not hesitate to contact us for additional details on our study. We thank you in advance for your collaboration and we look forward to hearing from you.

Best regards,
The Covid-NMA Team

**Methods S3: Data extraction form**

1. Reviewer (select one)

OP

TO

1. Type of intervention assessed in the study

Lopinavir/ritonavir

Other antiretroviral

Non-specific antiviral (interferons, IL2, immunoglobulin)

Broad-spectrum antiviral (umifenovir/ribavirin/favipiravir)

Hydroxychloroquine (with/without azithromycin)

Other antimalarial

Anticoagulants

Monoclonal antibodies

Corticosteroids

Immunosuppressant

Kinase inhibitors

Convalescent plasma

Mesenchymal stem cells

Social distancing/lockdown/travel restriction

Other policy non-pharmacologic intervention (mandatory BCG vaccination/mask use/

Other (specify: __________)

1. Country of affiliation for the corresponding author (if many sources, take this from the preprint V1): ________________________
2. Preprint DOI: __________________
3. Article DOI: _________________
4. Are the authors willing to share their data (preprint):

Yes, upon request

Yes, publicly available dataset

No

Unreported/unclear

1. Are the authors willing to share their data (article):

Yes, upon request

Yes, publicly available dataset

No

Unreported/unclear

1. Is the Abstract conclusion in the preprint (version 1):

Positive

Neutral

Negative

1. Does the preprint version 1 conclusion report uncertainty?

Yes, it reports uncertainty (e.g., The treatment may / could potentially lead to symptom improvement / Further studies are needed to confirm these findings)

No, it does NOT report uncertainty (e.g., The treatment leads to symptom improvement / should be administered to patients with Covid-19)

1. Is the Abstract conclusion in the preprint (latest version):

Positive

Neutral

Negative

1. Does the preprint (latest version) conclusion report uncertainty:

Yes

No

1. Is the article conclusion:

Positive

Neutral

Negative

1. Does the article conclusion report uncertainty?

Yes

No

1. Is there a change in effect size (HR/OR/risk ratio/event rate/regression coefficient) or in significance for any outcome, between the preprint (first version) and the preprint (latest version)?

Yes

No

1. Is there a change in effect size (HR/OR/risk ratio/event rate/regression coefficient) or in significance for any outcome, between the preprint (first version) and the journal article?

Yes

No

1. Is there a change in effect size (HR/OR/risk ratio/event rate/regression coefficient) or in significance for any outcome, between the preprint (latest version) and the journal article?

Yes

No

1. Is there a change in any of the following, that could alter the assessment of risk of bias, between the preprint (first version) and the preprint (latest version)?

Change in the description of the randomization (randomization process or allocation concealment)

Change in blinding (for clinicians or participants)

Change in blinding (for outcome assessors)

Change in missing data handling or proportion

Change in the selection of participants into the study

Change in the confounders adjusted for or in the adjustment method used

1. Is there a change in any of the following, that could alter the assessment of risk of bias, between the preprint (first version) and the journal article?

Change in the description of the randomization (randomization process or allocation concealment)

Change in blinding (for clinicians or participants)

Change in blinding (for outcome assessors)

Change in missing data handling or proportion

Change in the selection of participants into the study

Change in the confounders adjusted for or in the adjustment method used

1. Is there a change in any of the following, that could alter the assessment of risk of bias, between the preprint (latest version) and the journal article?

Change in the description of the randomization (randomization process or allocation concealment)

Change in blinding (for clinicians or participants)

Change in blinding (for outcome assessors)

Change in missing data handling or proportion

Change in the selection of participants into the study

Change in the confounders adjusted for or in the adjustment method used

Additional information:

To code a single data-sharing variable per study for the final analyses, when both the preprint and journal article were available, we compared the two data-sharing statements. If the two statements were in agreement or if the discrepancy was between “publicly available” and “upon request”, we kept the data-sharing statement reported in the preprint. When there was a disagreement: 1) if the preprint data-sharing was unclear/unreported, but journal-article data-sharing was reported, we took the article data-sharing statement; and 2) if the journal-article data-sharing was unclear/unreported, but preprint data-sharing was reported, we took the preprint data-sharing statement.

**Table S1: Sensitivity analysis of change in evidence components using important change in study results ^a^**

|  | **Overall sample (n = 139) ^c^** | | | | **COVID-NMA subgroup (n = 25) ^b^** | | | | |
| --- | --- | --- | --- | --- | --- | --- | --- | --- | --- |
|  | **Change between or within at least one evidence source pair (n = 139)** | First to latest preprint version  (n = 91) | First preprint version to journal article  (n = 66) | Latest preprint version to journal article  (n = 18) | **Change between or within at least one evidence source pair (n = 25)** | First to latest preprint version  (n = 18) | First preprint version to journal article (n = 15) | Latest preprint version to journal article (n = 7) |  |
| **Change in at least 1 evidence component** | **55 (40)** | 31 (34) | 30 (45) | 5 (28) | **13 (52)** | 7 (39) | 9 (60) | 1 (14) |  |
| Important change in study results | **29 (21)** | 17 (19) | 16 (24) | 3 (17) | **5 (20)** | 3 (17) | 4 (27) | 1 (14) |  |
| Change in risk of bias assessment | **-** | - | - | - | **5 (20)** | 1 (5) | 4 (27) | 1 (14) |  |
| Change in abstract conclusion | **33 (24)** | 18 (20) | 19 (29) | 3 (17) | **8 (32)** | 5 (28) | 5 (36) | 1 (14) |  |

^a^ We considered a change in study results among evidence sources to be important if 1) it represented an increase or decrease by ≥ 10% of the initial value in any effect estimate and/or 2) it led to a change in the p-value crossing the threshold of 0.05, for any study outcome.

^b^ Includes studies used in quantitative evidence synthesis and GRADE development in the COVID-NMA project (randomized controlled trials; interrupted time-series, non-randomized studies using causal inference analysis or multivariable regression adjustment, including at least 150 incident users), with multiple evidence sources or evidence source versions. For more information see: <https://covid-nma.com/emulated/>.

**^c^** Includes the studies in the COVID-NMA subgroup.

## Included

**Table S2. Usage data for preprint–journal article pairs in the complete sample (n = 66) and excluding evidence sources in which data usage equaled 0 at each estimate. ^a,b^**

|  | **Complete sample (n = 66)** | | **Excluding null values** | |
| --- | --- | --- | --- | --- |
|  | Preprint | Journal article | Preprint | Journal article |
| **Altmetric Attention Score^c^** | 0.6 [0.1–2.3] | 0.8 [0.1–4.2] | 0.7 [0.2–2.7] | 1.1 [0.2–5.0] |
| **PubPeer comments^d^** | 0.0 [0.0–0.0] | 0.0 [0.0–0.0] | 0.0 [0.0–0.0] | 0.0 [0.0–0.0] |
| **Citation count^c^** | 0.0 [0.0–0.1] | 0.1 [0.0–0.4] | 0.0 [0.0–0.1] | 0.1 [0.0–0.4] |

Data are median (IQR)

^a^ Data extracted 21 October 2020. Raw value divided by the number of days elapsed since online publication. The date the DOI was created according to publication metadata obtained from the Crossref API and was considered as the date of online publication.

^b^ Usage data could not be retrieved for 1 preprint that had no DOI. Missing values have not been imputed.

^c^ Source: Dimensions database (<https://www.dimensions.ai>).

^d^ Source: PubPeer platform (<https://pubpeer.com/>).

**Figure S1: Distribution of usage data in preprint–journal article pairs**


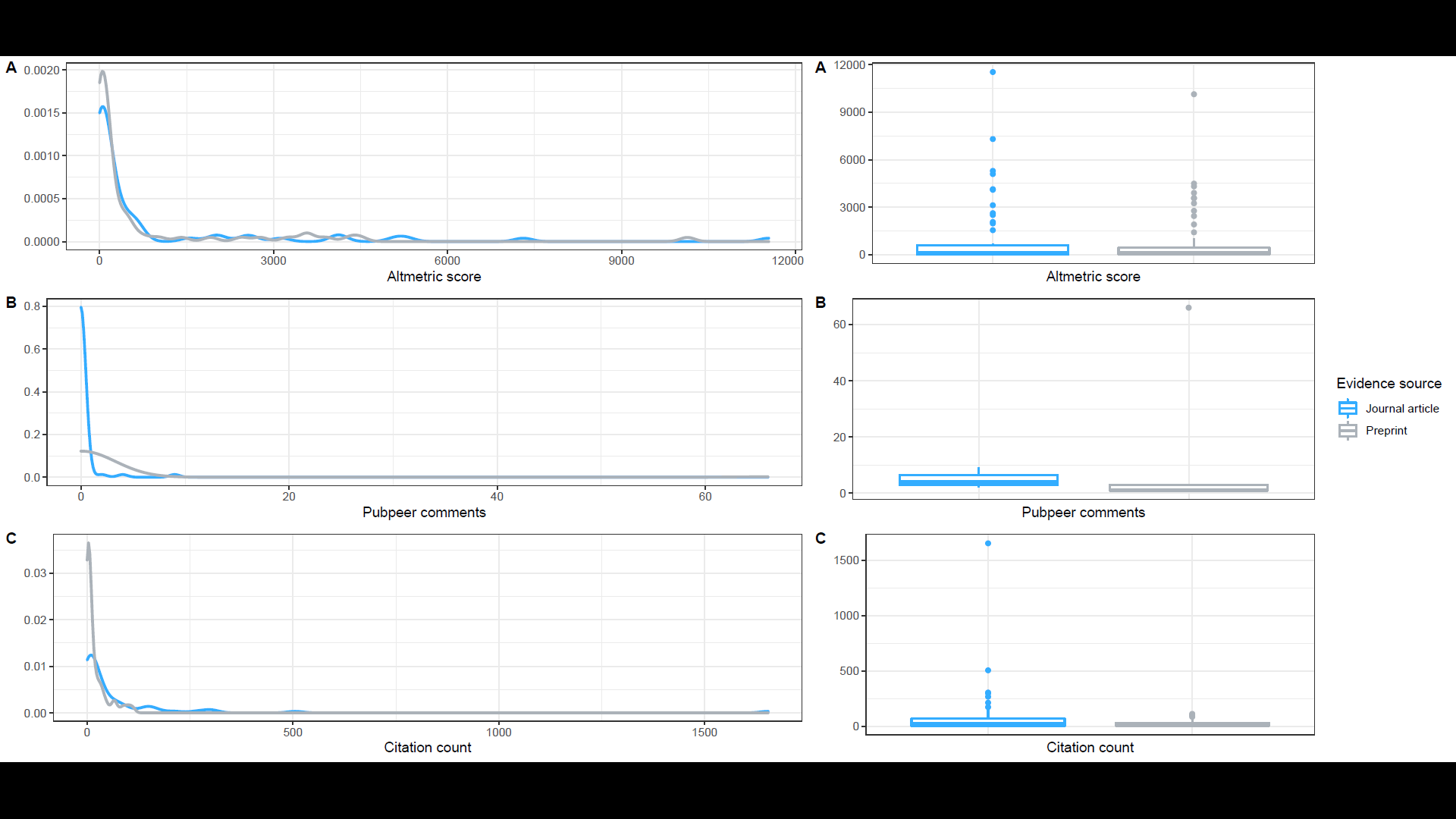


Each panel presents the distribution of usage data for one of three metrics: Altmetric Attention Score, PubPeer comments, and citations. The density plots on the left show the distribution of usage data for the preprints (in gray) and journal articles (in blue) for 66 studies that were reported in both evidence sources. The boxplots on the right show the median and interquartile range for the same studies, excluding evidence sources in which the data usage equals 0 at each plot. Despite earlier publication of preprints, journal articles are more widely disseminated. Data were extracted on 21 October 2020 and normalized for time (raw value divided by the number of days elapsed since online publication). For 1 preprint, usage data could not be retrieved
